# COVID-19 in 2 Persons with Mild Upper Respiratory Tract Symptoms on a Cruise Ship, Japan 

**DOI:** 10.3201/eid2606.200452

**Published:** 2020-06

**Authors:** Takeshi Arashiro, Keiichi Furukawa, Akira Nakamura

**Affiliations:** Asahi General Hospital, Chiba, Japan

**Keywords:** coronavirus, COVID-19, severe acute respiratory syndrome coronavirus 2, SARS-CoV-2, respiratory infections, PCR, cruise ship, travelers health, viruses, global health, Japan

## Abstract

We describe 2 cases of coronavirus disease in patients with mild upper respiratory symptoms. Both patients worked on a cruise ship quarantined off the coast of Japan. One patient had persistent, low-grade upper respiratory tract symptoms without fever. The other patient had rapid symptom cessation but persistent viral RNA detection.

Since December 2019, an outbreak of coronavirus disease (COVID-19) caused by severe acute respiratory syndrome coronavirus 2 (SARS-CoV-2) has been spreading globally (*1*). On January 25, 2020, a passenger disembarked a cruise ship in Hong Kong and on February 1 tested positive for SARS-CoV-2 (*2*). The ship docked in Yokohama, Japan, on February 3 for quarantine and isolation. On February 7, passengers and crew were provided thermometers and asked to check their temperature several times daily. Crew members were instructed to continue duties, report fever or respiratory symptoms, and follow quarantine instructions.

By February 28, a total of 705 COVID-19 cases were confirmed among 4,061 passengers and crew tested; 392 cases were asymptomatic, 36 persons were admitted to intensive care units, and 6 patients died (*2*). All case-patients from the ship were transferred to designated medical institutions in Japan (*3*).

Preliminary reports describe COVID-19 manifesting as pneumonia (*4*–*6*), but most cases are milder and could have more transmission potential because patients might not seek medical attention (*7*). Because of the lower threshold for testing persons on board, the cruise ship created an opportunity to observe mild COVID-19 cases and monitor patient symptoms. We describe 2 COVID-19 cases in persons with mild upper respiratory symptoms. The patients provided written, informed consent to share their clinical details.

Case 1 occurred in a 35-year-old woman from South Asia who worked as a restaurant server on the ship. On day 1 of her illness, February 7, she experienced throat dryness and a slight cough (Figure, panel A). She and her roommate shared a bathroom with 2 others who had similar symptoms earlier. Case-patient 1 reported her symptoms but continued to work because she was afebrile. On day 3, she had throat soreness, stayed in her room, and was tested for SARS-CoV-2 by reverse transcription PCR (RT-PCR). On days 4–5, her symptoms diminished. On day 6, she was told she tested positive and was transferred to Asahi General Hospital (Chiba, Japan). At admission, she had a slight sore throat and cough. Her temperature was 36.5°C; blood pressure, 113/85 mm Hg; pulse, 92 bpm; respiration, 16 breaths/min; and oxygen saturation, 95% on ambient air. She had no underlying medical conditions and was taking no routine medication. On examination, her throat was bright red without exudates, lung auscultation was clear, and chest radiographs and blood tests were not clinically significant (Appendix Figure 1, panel A, Table 1). We did not suspect pneumonia and did not perform computed tomography. On day 8, she reported slight rhinorrhea. On day 9, RT-PCR again was positive for SARS-CoV-2. Her symptoms continued to diminish, and by day 10 she had discontinued all medications. RT-PCR results were positive on days 13 and 15, negative on day 19, positive again on day 20, and negative again on days 22 and 23, meeting the criteria for discharge, 2 consecutive negative assays. She never had fever, shortness of breath, or sputum, and daily lung auscultation was clear, suggesting absence of pneumonia.

**Figure:**
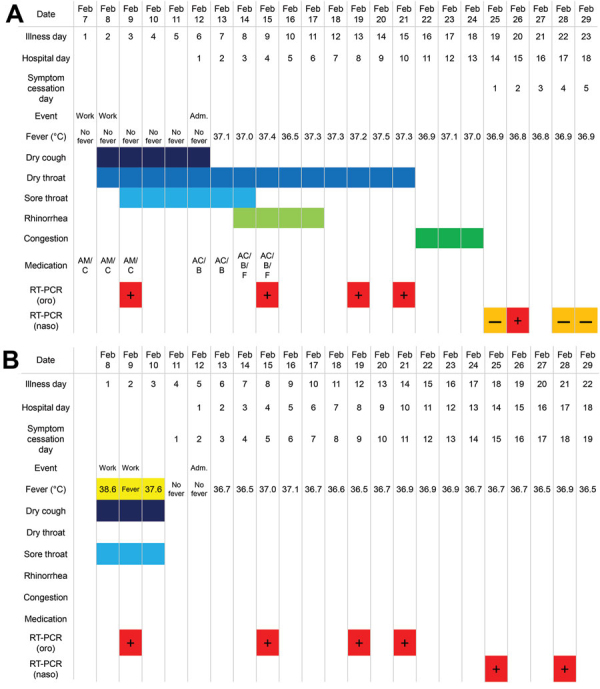
Clinical courses of 2 case-patients with 2019 novel coronavirus (COVID-19) admitted from a cruise ship docked in Japan, 2020. A) Case-patient 1, a 35-year-old female who worked on the ship as a restaurant server. B) Case-patient 2, a 27-year-old male who worked on the ship as a kitchen cleaner. Acetaminophen was administered on an as-needed basis >2×/day after taking body temperature, so measured body temperature is not affected. Nasopharyngeal swabs were used after February 21, 2020, because a study by Zou et al. (*10*) suggested higher sensitivity of nasopharyngeal swab specimens over oropharyngeal swab specimens. AC, acetaminophen; Adm., admission; AM, amoxicillin; B, bakumondoto, a multiherb kampo medicine for dry cough; C, codeine-containing cough syrup; F, fexofenadine; naso, nasopharyngeal swab; oro, oropharyngeal swab; RT-PCR, reverse transcription PCR.

Case 2 occurred in a 27-year-old man from South Asia who worked as a kitchen cleaner on the ship. On day 1 of his illness, February 8, he had a fever (38.6°C), sore throat, and cough. His roommate had similar symptoms that started 2 days before. Case-patient 2 reported his symptoms but continued to work. On day 2, he was tested for SARS-CoV-2 but continued to work. On day 3, his fever persisted, so he stayed in his room. By day 4, his symptoms resolved. On day 5, he was told he tested positive for SARS-CoV-2 and was transferred to Asahi General Hospital. At admission, he had no respiratory or other symptoms. He had no underlying medical conditions and was taking no routine medication. His temperature was 36.0°C; blood pressure, 132/85 mm Hg; pulse, 87 bpm; respirations, 16 breaths/min; and oxygen saturation, 95% on ambient air. On examination, his throat was bright red without exudates (Appendix Figure 2), lung auscultation was clear, and chest radiographs and blood tests were not clinically significant (Appendix Figure 1, panel B, Table 2). He remained asymptomatic, but on day 8, RT-PCR results were positive; results remained positive on days 12, 14, 18, and 21. His throat redness did not improve, but he did not report throat soreness or discomfort. He never experienced shortness of breath or sputum, and daily lung auscultations were clear.

We describe 2 mild cases of COVID-19 without discernable pneumonia, which could represent the clinical course in young, healthy persons. Worldwide, cases are appearing without apparent epidemiologic links (*8*). As the virus spreads, more mild COVID-19 cases are likely, and clinicians should be aware of clinical manifestations in the absence of severe symptoms. Case-patient 2’s symptoms rapidly decreased, but detectable viral RNA persisted for >2 weeks. As of February 27, patients in Japan must have 2 consecutive negative RT-PCR results before they can be discharged (*9*). Viral RNA detection does not necessarily indicate infectivity, so we urgently need guidance for detection and management of mild COVID-19 to prepare for a possible pandemic and avoid overwhelming healthcare systems.

## Addendum

As of April 6, a total of 712 coronavirus disease cases had been confirmed among 3,711 passengers and crew of the cruise ship (19.2%) (https://www.mhlw.go.jp/stf/houdou/houdou_list_202004.html). At the time of testing, 410 (57.6%) were asymptomatic; of those, 79 (11.1%) had symptoms develop within the 14-day self-monitoring period, leaving 331 persons who tested positive and remained asymptomatic after the self-monitoring period (46.5% of all cases). Forty (5.6%) patients were admitted to intensive care units (ICUs); 12 patients died (1.7%), including 1 person reported by the government of Australia to have died after transferring home. Of the cases we reported, case-patient 2 remained asymptomatic; RT-PCR results were negative on day 24, positive again on day 25, and negative again on days 27 and 28, meeting the criteria for discharge. His throat redness had improved by discharge. By March 27, both patients had been sent back to their home country by air and instructed to self-isolate at home for an additional 14 days.

Appendix Additional information on 2 cases of Coronavirus disease 2019 (COVID-19) with mild upper respiratory symptoms contracted in a cruise ship off the coast of Japan.
