# Supplementary material for: COVID-19 in 2 Persons with Mild Upper Respiratory Tract Symptoms on a Cruise Ship, Japan
Source: Emerg Infect Dis. 2020 Jun;26(6):1345–8. doi: 10.3201/eid2606.200452 (PMC7258480; doi:10.3201/eid2606.200452)
Supplement: Appendix — Additional information on 2 cases of Coronavirus disease 2019 (COVID-19) with mild upper respiratory symptoms contracted in a cruise ship off the coast of Japan. [file 20-0452-Techapp-s1.pdf]

Article DOI: https://doi.org/10.3201/eid2606.200452

COVID-19 in 2 Persons with Mild Upper Respiratory Symptoms on a Cruise Ship, Japan

Appendix

Appendix Table 1. Clinical laboratory results at time of hospital admission, day 6 post illness onset, for case 1 of 2019 novel coronavirus (COVID-19) diagnosed in a woman who worked on a cruise ship

Measure	Patient values	Reference range
Leukocyte count/µL	3,900	3,300–8,600
Red blood cell count/µL	5,210,000*	3,860,000–4,920,000
Neutrophils, %	56.9	38.3–74.7
Lymphocytes, %	32.1	21.2–51.0
Monocytes, %	6.0	2.7–8.0
Eosinophils, %	4.7	0.2–8.4
Basophils, %	0.3	0.2–2.0
Absolute neutrophil count/µL	2,219	
Absolute lymphocyte count/µL	1,252	
Hemoglobin, g/dL	14.6	11.6–14.8
Hematocrit, %	43.0	35.1–44.4
Platelets/µL	159,000	158,000–348,000
Sodium, mmol/L	140	138–145
Potassium, mmol/L	3.9	3.6–4.8
Chloride, mmol/L	106	101–108
Calcium, mg/dL	9.4	8.8–10.1
Phosphate, mg/dL	3.8	2.7–4.6
Total protein, g/dL	7.7	6.6–8.1
Albumin, g/dL	4.5	4.1–5.1
Alanine aminotransferase, U/L	18	7–23
Aspartate aminotransferase, U/L	17	13–30
Gamma-glutamyl transpeptidase, U/L	17	9–32
Total bilirubin, mg/dL	3.2	0.4–1.5
Amylase, mmol/L	67	44–132
Blood urea nitrogen, mg/dL	6†	8–20
Creatinine, mg/dL	0.56	0.46–0.79

Measure	Patient values	Reference range
Lactate dehydrogenase, U/L	181	124–222
Creatine kinase, U/L	69	41–153
Glucose, mg/dL	112*	73–109
Hemoglobin A1c, %	5.6	4.9–6.0
C-reactive protein, mg/dL	0.12	0.0–0.14
Prothrombin time, s	11.9	
International normalized ratio	0.94	
Activated partial thromboplastin time, s	31.5	25–35
Fibrinogen, mg/dL	250	220–410
Antistreptolysin O, IU/mL	97	<239

*Value is above the reference range.

Appendix Table 2. Clinical laboratory results at time of hospital admission, day 7 post illness onset, for case 2 of 2019 novel coronavirus (COVID-19) diagnosed in a man who worked on a cruise ship

Measure	Patient values	Reference range
Leukocyte count/μL	5,200	3,300–8,600
Red blood cell count/μL	6,490,000*	4,350,000–5,550,000
Neutrophils,%	41.7	38.3–74.7
Lymphocytes, %	44.1	21.2–51.0
Monocytes, %	9.6*	2.7–8.0
Eosinophils, %	3.1	0.2–8.4
Basophils, %	1.5	0.2–2.0
Absolute neutrophil count/μL	2,168	
Absolute lymphocyte count/μL	2,293	
Hemoglobin, g/dL	15.9	13.7–16.8
Hematocrit, %	48.4	40.7–50.1
Platelets/μL	291,000	158,000–348,000
Sodium, mmol/L	139	138–145
Potassium, mmol/L	3.9	3.6–4.8
Chloride, mmol/L	105	101–108
Calcium, mg/dL	9.5	8.8–10.1
Phosphate. mg/dL	2.2†	2.7–4.6
Total protein, g/dL	7.7	6.6–8.1
Albumin, g/dL	4.7	4.1–5.1
Alanine aminotransferase, U/L	41	10–42
Aspartate aminotransferase, U/L	27	13–30
Gamma-glutamyl transpeptidase, U/L	57	13–64
Total bilirubin, mg/dL	0.4	0.4–1.5
Amylase, mmol/L	97	44–132
Blood urea nitrogen, mg/dL	11	8–20
Creatinine, mg/dL	0.84	0.46–0.79

Measure	Patient values	Reference range
Lactate dehydrogenase, U/L	170	124–222
Creatine kinase, U/L	166	41–153
Glucose, mg/dL	115*	73–109
Hemoglobin A1c, %	6.0	4.9–6.0
C-reactive protein, mg/dL	0.06	0.0–0.14
Prothrombin time, s	11.6	
International normalized ratio	0.92	
Activated partial thromboplastin time, s	31.5	25–35
Fibrinogen, mg/dL	258	220–410
Antistreptolysin O, IU/mL	69	<239

*Value is above reference range.

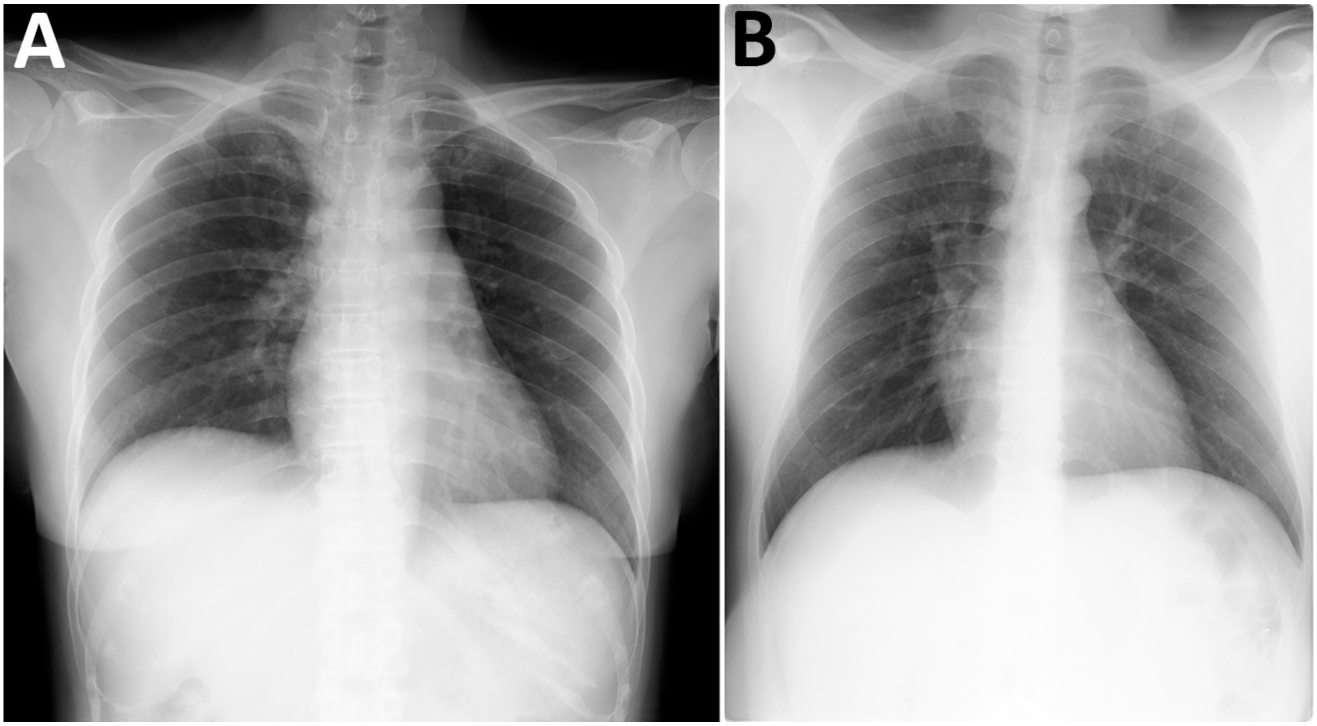

Appendix Figure 1. Anteroposterior chest radiographs of 2 patients with mild upper respiratory symptoms who tested positive for 2019 novel coronavirus (COVID-19). A) Case-patient 1 at day 6 post illness onset. B) Case-patient 2 at day 7 post illness onset.

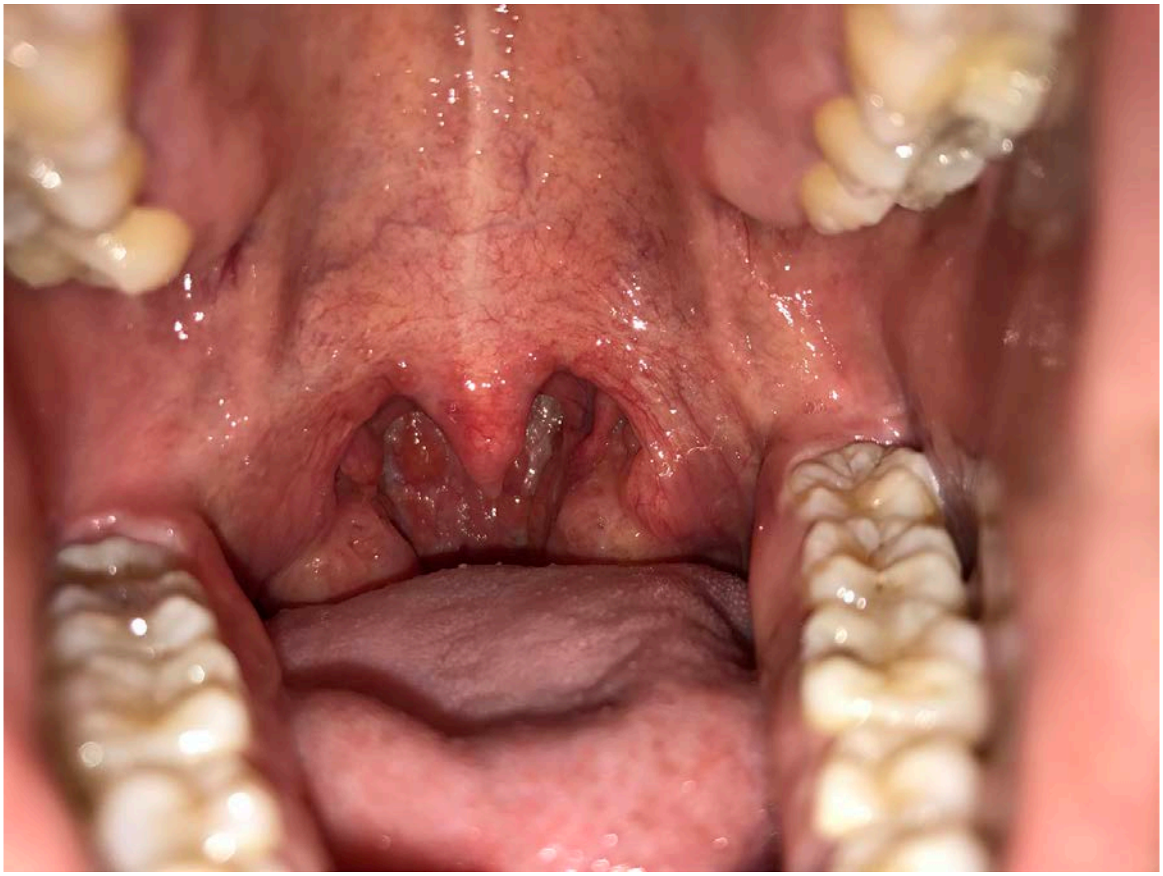

Appendix Figure 2. Throat redness of case-patient 2, a 27-year-old man with mild upper respiratory symptoms who tested positive for 2019 novel coronavirus (COVID-19).
